# Prioritizing Clinically Relevant Copy Number Variation from Genetic Interactions and Gene Function Data

**DOI:** 10.1371/journal.pone.0139656

**Published:** 2015-10-05

**Authors:** Justin Foong, Marta Girdea, James Stavropoulos, Michael Brudno

**Affiliations:** 1 Department of Computer Science, University of Toronto, Toronto, Ontario, Canada; 2 Hospital of Sick Children, Toronto, Ontario, Canada; University of Illinois at Chicago, UNITED STATES

## Abstract

It is becoming increasingly necessary to develop computerized methods for identifying the few disease-causing variants from hundreds discovered in each individual patient. This problem is especially relevant for Copy Number Variants (CNVs), which can be cheaply interrogated via low-cost hybridization arrays commonly used in clinical practice. We present a method to predict the disease relevance of CNVs that combines functional context and clinical phenotype to discover clinically harmful CNVs (and likely causative genes) in patients with a variety of phenotypes. We compare several feature and gene weighing systems for classifying both genes and CNVs. We combined the best performing methodologies and parameters on over 2,500 Agilent CGH 180k Microarray CNVs derived from 140 patients. Our method achieved an F-score of 91.59%, with 87.08% precision and 97.00% recall. Our methods are freely available at https://github.com/compbio-UofT/cnv-prioritization. Our dataset is included with the supplementary information.

## 1 Introduction

Remarkable improvements in genotyping methods have arisen recently, including microarrays and more recently High Throughput Sequencing (HTS) technologies. These new technologies allow for inexpensive and accurate identification of millions of genetic variants, including Single Nucleotide and Copy Number Variants (SNVs and CNVs) present in any human. Now large-scale studies of human populations are possible, such as the Wellcome Trust Case-Control Study [1] and the 1000 Genomes Project [2]. However, the clinical use of whole-genome genotyping, whether array or sequencing-based, has been hindered by the difficulty of identifying disease-causing variants amongst the millions of benign differences between human genomes. A molecular diagnostic lab analyzes the DNA of individuals with suspected genetic disorders to uncover the molecular cause of the disease. Depending on the disorder, the individual may undergo screening for a small number of relevant genes, or whole-genome analysis. In either case, the discovered variants are presented to a molecular geneticist who manually analyzes each variant and determines whether it is likely to be involved in the disorder. In many cases, harmful variants must be identified solely based on the patient’s clinical phenotype (description of symptoms) rather than a named disorder, as a large fraction of individuals presented to a molecular diagnostic facility cannot be easily given a specific diagnosis. Because the predominant fraction of the variants found in any human’s genome do not contribute to the patients clinical phenotype and are likely benign [3], software tools for harmful variant prioritization [4, 5] play an important role in assisting the geneticist. However, these methodologies typically only address single nucleotide variation, and rarely have the specificity or sensitivity to be used without human supervision [6].

In this work we develop methodologies to assist in the prediction of the harmfulness of large-scale CNVs. These variants can significantly alter cellular processes by simultaneously deleting or duplicating multiple genes, all with distinct functions [7]. Identification of clinically harmful CNVs is significantly more complicated than single nucleotide variants (SNVs). Conservation, impact on splicing or protein structure, or location within a known functional element have been shown to closely correlate to harmfulness [4, 8] for SNVs. However, such features are less informative for larger CNVs: while the conservation level of a smaller variant is indicative of its functionality, all larger CNVs are almost certain to contain multiple conserved regions, and conversely, it has been shown that the deletion of some highly conserved regions leads to no discernible phenotype [9]. Even the approach of filtering variants that are present at high frequency in the human population has important caveats when working with CNV data from comparative genome hybridization (CGH) arrays. The breakpoints of most CNVs are not determined precisely, and multiple partially overlapping variants from several individuals may or may not be identical.

To date, most efforts to analyze the medical impact of CNVs have concentrated on well-matched cases and controls. For example, by carefully analyzing the CNV loci and the corresponding pathways, researchers have been able to identify novel genes involved in complex disorders, such as Autism [10]. One recent study also revealed the existence of physical interactions between different genes harboring causal mutations for several immune-mediated disorders, including rheumatoid arthritis and Crohn’s disease [11]. Both of these approaches, however, aim to identify disease genes, rather than discover the medically relevant CNVs within each patient.

A prioritization pipeline for CNVs in patients with intellectual disability is proposed in [12]. Their approach considered, for each CNV, a number of genomic parameters, such as gene density, length, repeat content of the CNV, as well as some functional features, such as region annotation in specific KEGG pathways [13] or MGI phenotypes [14]. They found that the best predictors of harmfulness of CNVs were mouse phenotypes and the density of LINE repeats in the variant, yet they made no attempt to correlate the harmful CNVs to the patient phenotypes through gene function, or to identify which gene(s) within the larger CNV actually contribute to the phenotype.

The methods developed in the current manuscript take into consideration the mutation’s broader molecular and functional context, as well as the phenotype of the patient in whom it was observed. Our work develops upon several recent studies [15–17], that propose approaches for prioritizing disease genes which combine multiple sources of information, including phenotype-phenotype and phenotype-gene associations, as well as gene/protein interaction networks. Unlike our approach, however, these methods utilize phenotype-gene correlations to assist the prioritization of genes for a specific disease, rather than for discovering clinically harmful genes (and CNVs) in patients with a variety of disorders. A method that explicitly allows for multiple disorders is [18], which uses a cross-species phenotype ontology to determine phenotypic features related to a query CNV. In addition to identifying if a CNV is causative, it also prioritizes which genes affected by the CNV are relevant. Finally previous work that is perhaps most closely related to our methodologically identifies CNV disrupted pathways through label propagation on a protein-protein interaction network [19].

Our algorithm combines patient phenotype information, phenotype-gene associations as well as gene interaction networks to prioritize the results. We show that incorporating this information leads to a substantial improvement in recall. We achieved an F-score of 91.59%, with 87.08% precision and 97.00% recall on our dataset of 2,643 CNVs (including 162 harmful ones) derived from 140 patients genotyped with Agilent CGH 180k Microarrays and characterized as benign or harmful by a molecular geneticist.

## 2 Background

Our model is built upon a variety resources and tools allowing for the interpretation of our data across multiple layers of knowledge. These layers range from disease and phenotype information and relationships to disease-causative genes, gene functions and interactions. Their interoperability is ensured by the use of standardized vocabularies and identification systems.

### 2.0.1 HPO

The Human Phenotype Ontology (HPO) [20], aims to provide a standardized vocabulary for the phenotypic abnormalities (approximately 10,000 terms) encountered in human disease, and the semantic relationships between them (http://www.human-phenotype-ontology.org/). Phenotypes within HPO are commonly annotated with hereditary disorders, with > 50,000 crosslinks available between HPO and the Online Mendelian Inheritance in Man (OMIM) [21] database.

Each patient description consists of a set of HPO terms, thus we benefit from the knowledge in these annotations, allowing us to access mappings between phenotypes and diseases or even known causative genes.

### 2.0.2 GO and GOA

The Gene Ontology (GO) project [22] (http://www.geneontology.org/) provides structured controlled vocabularies and classifications. The GO covers several domains of molecular and cellular biology and are freely available for community use in the annotation of genes, gene products and sequences. Using this extensive vocabulary for describing gene functions, the Gene Ontology Annotation (GOA) database [23] (http://www.ebi.ac.uk/GOA) consists of high-quality electronic and manual annotations to the UniProt Knowledgebase (Swiss-Prot, TrEMBL and PIR-PSD). GOA associates meta-information to each of the available gene annotations, including their evidence sources, thus allowing for advanced filtering for the most relevant and reliable annotations.

Because of the extensive detail of available GO terms (> 35,000), it is common practice to limit the number of available terms by making a “cut” across the ontology. We keep terms above this cut, while mapping terms below to their nearest available ancestor. In this work we use GO Slim [22] to reduce the number of possible GO terms to 105.

In our model, we use GO, GO-Slim, and GOA extensively for describing CNV affected genes by their GO functions. Such a detailed functional profile facilitates the discovery of patterns among the genes suspected to be causative for specific phenotypes.

### 2.0.3 Semantic similarity in ontologies

Both HPO and GO are ontologies structured as *taxonomies* where concepts are organized into broader concept categories via the “*is a*” relationship. Furthermore, concepts defined by HPO and GO annotate other entities, such as OMIM diseases and human genes respectively. The “*is a*” relation can be used for defining similarities between the concepts, based on their specificity within the annotations. The other relations are not used for defining similarities.

The evaluation of semantic similarity in a taxonomy using *information content* has been discussed in several papers, including [24] for generic taxonomies annotating generic targets and [25] for the specific case of HPO with OMIM annotations in the context on differential diagnosis. The approach, briefly explained below, is defined for any taxonomy of concepts that annotate a set of targets. In our case, the taxonomy can be either GO with gene names as targets, or HPO with OMIM disorders as targets.

Let *T* = (*V*, *R*) be a directed acyclic graph representing the taxonomy, where *V* is the set of terms (concepts) and *R* = {(*u*, *v*), *u*, *v* ∈ *V*} defines the parent-child relations within the taxonomy. Let *A* = (*N*, *C*, *E*) be the bipartite graph representing the available annotations, where *N* is the set of target nodes, *C* ⊆ *V* is the set of concepts used to annotate the targets, and *E* = {(*c*, *n*)∣*c* ∈ *C*, *d* ∈ *D*} is the set of edges between *N* and *C*, designating mappings between targets and their annotations. Let *a*(*n*) be all the neighbors in *A* of a target *n*, namely all the concepts known to be associated *n*. We assume that, if (*c*, *n*) is an edge in *A*, then (*p*, *n*) is an edge in *A* for any *p* that is an ascendant of *c* in the taxonomy *T*; in other words, if a target is annotated with a concept, then we consider that all broader concepts which include it are also annotations of that target. The similarity between two sets of concepts *X* and *Y* depends on the annotated targets as follows [25]:

$$\begin{array}{c} sim\left(X,Y\right)=avg_{x\in X}\max_{y\in Y}IC(MICA(x,y)) \end{array}$$ (1)

where *MICA*(*x*, *y*) denotes the most informative (having the highest information content) common ancestor of terms *x* and *y* in the taxonomy *T*, and the information content *IC* of a taxonomy term *t* is computed with respect to the annotation graph *A* as

$$\begin{array}{c} IC\left(t\right)=-\log(freq_{A}\left(t\right)) \end{array}$$ (2)

designating as more informative those concepts which annotate fewer targets.

Our model uses this semantic similarity measure to determine closely related phenotypic manifestations, as well as very similar functional profiles of genes. Together with the knowledge comprised in gene-phenotype annotations, this similarity measure helps infer which genes are more likely to be causative for the observed phenotype.

### 2.0.4 Gene networks via GeneMANIA

Gene and protein interaction networks provide valuable insight regarding the roles genes play in complex diseases. To incorporate this information in our model, we rely on GeneMANIA [26]. GeneMANIA is a tool that uses multiple networks derived from different genomic or proteomic data sources to produce a single, composite functional association network with normalized interaction weights. We use these weights to build a network of “neighbor” genes for the genes affected by CNVs in our dataset. These neighbors are included in our decision process for inferring CNV harmfulness.

## 3 Results

In this section we present the performance of our methods on a dataset of CNVs detected in clinical patients with known phenotypes. These CNVs were evaluated by a clinical geneticist as harmful or benign. We choose an optimal weighing system for both gene ontology features and gene relevance to build a classifier for the harmfulness of a gene. We then show that this classifier, in combination with the CNV length and the frequency in DGV, achieves greater accuracy in predicting CNV harmfulness than any subset of the three information sources. The results presented here are obtained by aggregating the output of 20 runs on random training / testing splits generated as explained in Section 5.1.

### 3.1 Dataset

#### 3.1.1 Raw data

We have obtained Agilent CGH 180k Microarray dataset from the Molecular Diagnostic Laboratory and the Microarray Facility at the Hospital of Sick Children. This data set consists of 140 patient reports, with 162 harmful and 2,481 benign CNVs annotated by a molecular geneticist. Each of these patients was annotated with a set of phenotypes (HPO codes), and each had at least one harmful CNV. The majority of these patients (119) were annotated with the phenotype *developmental delay*, 10 patients presented *facial dysmorphism*, while 7 other phenotypes (*autism*, *cleft palate*, *congenital heart defect*, *failure to thrive*, *microcephaly*, *seizures*, *short stature*) are each assigned to 1 or 2 patients of our curated dataset.

#### 3.1.2 Pre-processing

We used BEDTools [27] to annotate the CNVs with Ensembl genes from UCSC’s table browser [28] for the hg18 Human assembly. Each gene was then labeled with Gene Ontology (GO) [22] functions using the Gene Ontology Annotation database (GOA) [23], resulting in > 3,000 potential features. To reduce the feature space we mapped each term to its nearest ancestor within Generic GO Slim [22], to obtain a set of 105 features (GO terms identifiers).

### 3.2 Optimal model weights

#### 3.2.1 Choosing a feature weighing system

**Fig 1:**
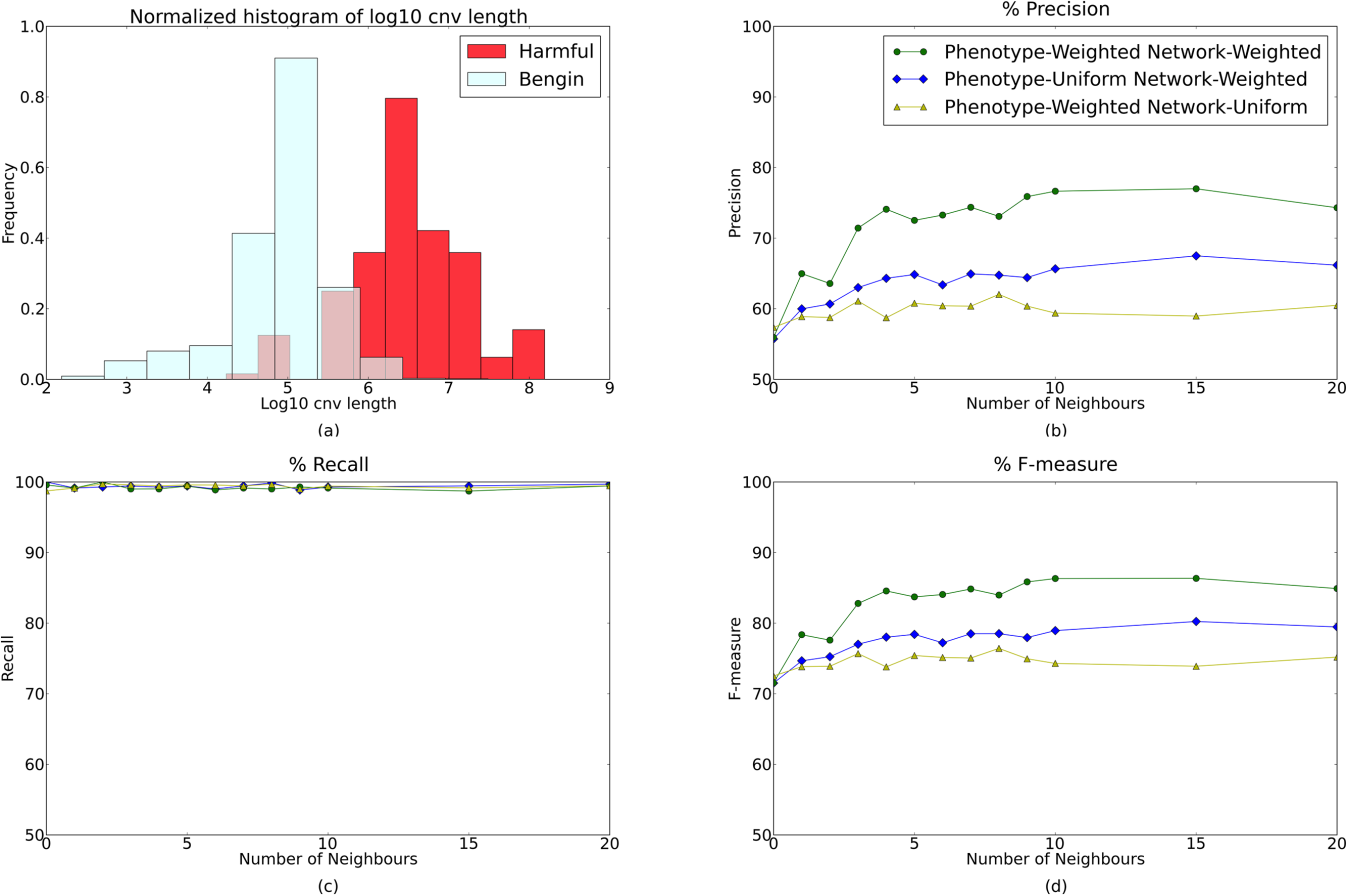
Importance of Model Features. (a) Histogram of CNV lengths (on log scale) for harmful and benign CNVs within our dataset shows that harmful CNVs are more likely to be longer, and hence likely affect more genes and gene functions. (b-d) Precision (b), recall (c) and f-measure (d) for predicting harmful versus benign CNVs relative to the number of closest neighbors considered within the gene interaction network. Both precision (b) and f-measure (d) improve as we expand the number of neighbors considered, but stabilize or slightly descend after 10 neighbors. We also see an improvement from utilizing the patient phenotypes uniform model in precision and accuracy as we add the ranking as a source for weighing our features.

We evaluate the performance of both feature weighing systems for CNV classification presented in Section 5.2.1 (*i.e*. **Network-Uniform** and **Network-Weighted**) and show the results in Fig 1b–1d.

As a first observation, all precision and accuracy curves (Fig 1b and 1d) show an ascending trend as we take into account more neighbors of the affected genes, with a tendency to stabilize or slightly descend after 10 neighbors. This shows the significance of using relevant gene interactions to infer gene functions affected by a mutation. However, interrogating too many such neighbors can result in uninformative or even noisy features being introduced in the model. We also observe an improvement over the Network-Uniform model in precision and F-measure when using the Network-Weighted model.

Note also that under both of these weighing systems the recall on harmful CNVs (Fig 1c) is nearly perfect. We consider this a significant advantage of our model: such a low false negative rate means it is very unlikely to “miss” a harmful variant (hence CNVs classified as benign need not be manually verified).

#### 3.2.2 Choosing a gene weighing system

The **Phenotype-Weighted** and **Phenotype-Uniform** models for weighing genes (training points for the gene classifier) that were described in Section 5.2.2 have been tested on our dataset, and the results are captured in Fig 1b–1d. As expected, gene classification accuracy improves with the introduction of the gene weights, since the model learns to focus on those GO terms that are consistent with prior knowledge regarding gene-phenotype associations. Similarly, CNV precision is boosted by these weights and continues to improve as we take into consideration the relevance of more close neighbors, without reducing the recall.

### 3.3 Performance on CNV prioritization

Among the different setups presented in sections 5.2.1 and 5.2.2, we have chosen the parameters which produce the best results on our data: (1) the **Network-Weighted model** for feature weighting, (2) the **Phenotype-Weighted**-based gene weighting, and (3) 10 closest neighbors for relevant GO term incorporation. As shown before in Fig 1 which summarizes the results, using these weights derived from gene interaction data and from known gene-phenotype associations leads to a precision of 76.65% and a 99.13% recall computed as the average over 20 runs.

**Fig 2:**
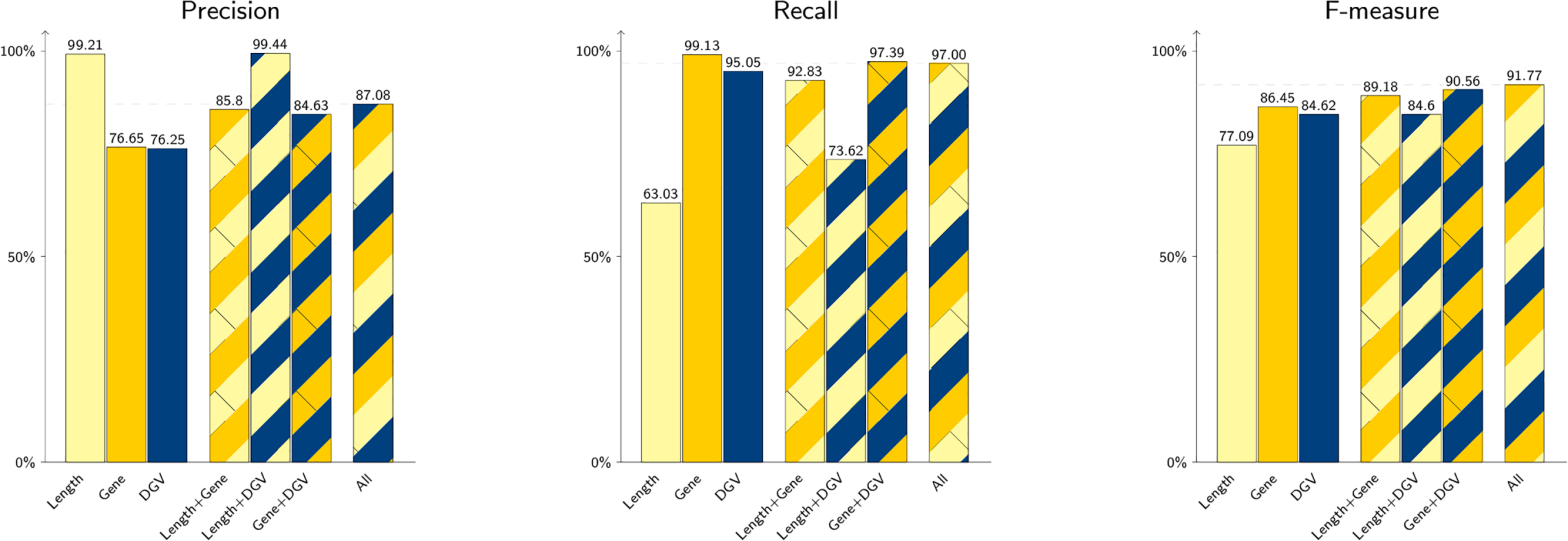
Precision, recall and f-measure for CNVs when combining the three following features length, DGV and gene. Length is the CNV length. DGV is a measure of the CNV’s frequency in the Database of Genomic Variants. Gene is the feature derived from the previous machine learning step in this method.

After choosing the best-performing parameters for our method, we compared it to two other simple classifiers that take into account the length of the CNV and its occurrences in DGV respectively, which are known to correlate well with the harmfulness of a CNV. The results of this comparison are depicted in Fig 2. Our gene-based method has a better recall (99.13%) than the DGV classifier (95.05%), and significantly outperforms the classification based solely on the CNV length (63.03%), yet the latter is more precise (99.21%). The overall accuracy (based on F-measure) shows the gene-based method as the best performing of the three.

Since the three sources of information for establishing CNV harmfulness have different strengths (e.g. our gene-based method has a very low false positive rate, while the length-based classification is very precise), we looked into combining them into a single classifier (described in Section 5.3) in order to achieve better accuracy. Fig 2 presents the performance of various combinations. For example, length and DGV-overlaps achieve together an F-measure of 84.06%, but adding the gene-harmfulness evidence obtained by our method will further improve the score up to 91.59%. To better understand this improvement, we evaluated our method on the CNVs that have been incorrectly classified by Length-DGV classifier, and obtained the correct labeling in 34 (92%) of the cases. This result emphasizes the ability of our method to capture correlations between gene information and CNV harmfulness that elude the classifier based on length and DGV overlaps.

To sum up the findings depicted in Fig 2, our approach for assessing CNV harmfulness based on gene and phenotype evidence outperforms the classifiers based on CNV length and DGV overlaps. Combining the gene feature along with the CNV length and DGV overlaps in a Naive Bayes model produces an F-score of 91.59%—the highest among all the evaluated models, with a 87.08% precision and a 97.00% recall measured on our dataset.

When testing the final classifier that combines all three features on an unbalanced dataset, where we test on whole patients and beign CNVs outnumber harmful ones by ∼ 15 : 1, we achieve an F-score of 64.29%, with a 48.07% precision and a 97.01% recall. Our recall remains high while the precision drops on an unbalanced dataset.

## 4 Discussion

Current advances in genotyping, including microarrays and High Throughput Sequencing (HTS) technologies, allow for inexpensive and accurate identification of the millions of genetic variants, including Single Nucleotide and Copy Number Variants (SNVs and CNVs) present in any human. However, the clinical use of whole-genome genotyping, whether array or sequencing-based, has been hindered by the difficulty of identifying disease-causing variants amongst the millions of differences between human genomes. Current methods utilize phenotype-gene correlations to assist the prioritization of genes for a specific disease, rather than for discovering clinically harmful genes (and CNVs).

In this paper we presented a method to combine functional context and phenotype to discover clinically harmful genes (and CNVs) in patients with a variety of disorders. Our results show that incorporating this information leads to a substantial improvement in recall. We achieved an F-score of 91.59%, with 87.08% precision and 97.00% recall on our dataset of Agilent CGH 180k Microarray CNVs. This high recall is a significant advantage of our model. It is unlikely to “miss” a harmful variant with such a low false negative rate so CNVs classified as benign will have a low priority of manual verification. This is highly desirable for a diagnostic test.

While the recall is near perfect, the precision could be improved. A natural extension to this method lies in the transition between the gene and CNV classification steps. In this paper, a simple “boolean or” is used to determine the CNV’s classification from its overlapping genes. This method considers any CNV with at least a single harmful gene to be classified as harmful and leads to an overcalling of harmful CNVs. Using a less simplistic method for combining the gene classifications such as the “noisy or” to weigh each gene by its confidence value may improve the precision.

## 5 Methods

This study was approved by the Hospital for Sick Children’s Research Ethics Board. All patient information was anonymized and de-identified prior to analysis.

Our method for classifying harmful CNVs relies on two orthogonal types of phenotypic and genetic data: **(1) phenotypic descriptions** of patients and the relationships between these phenotypes as specified by HPO, together with their non-exhaustive associations to disease genes (via OMIM), and **(2) genes affected by CNVs and gene interactions** (via GeneMANIA), with their respective functions according to GO.

**Fig 3:**
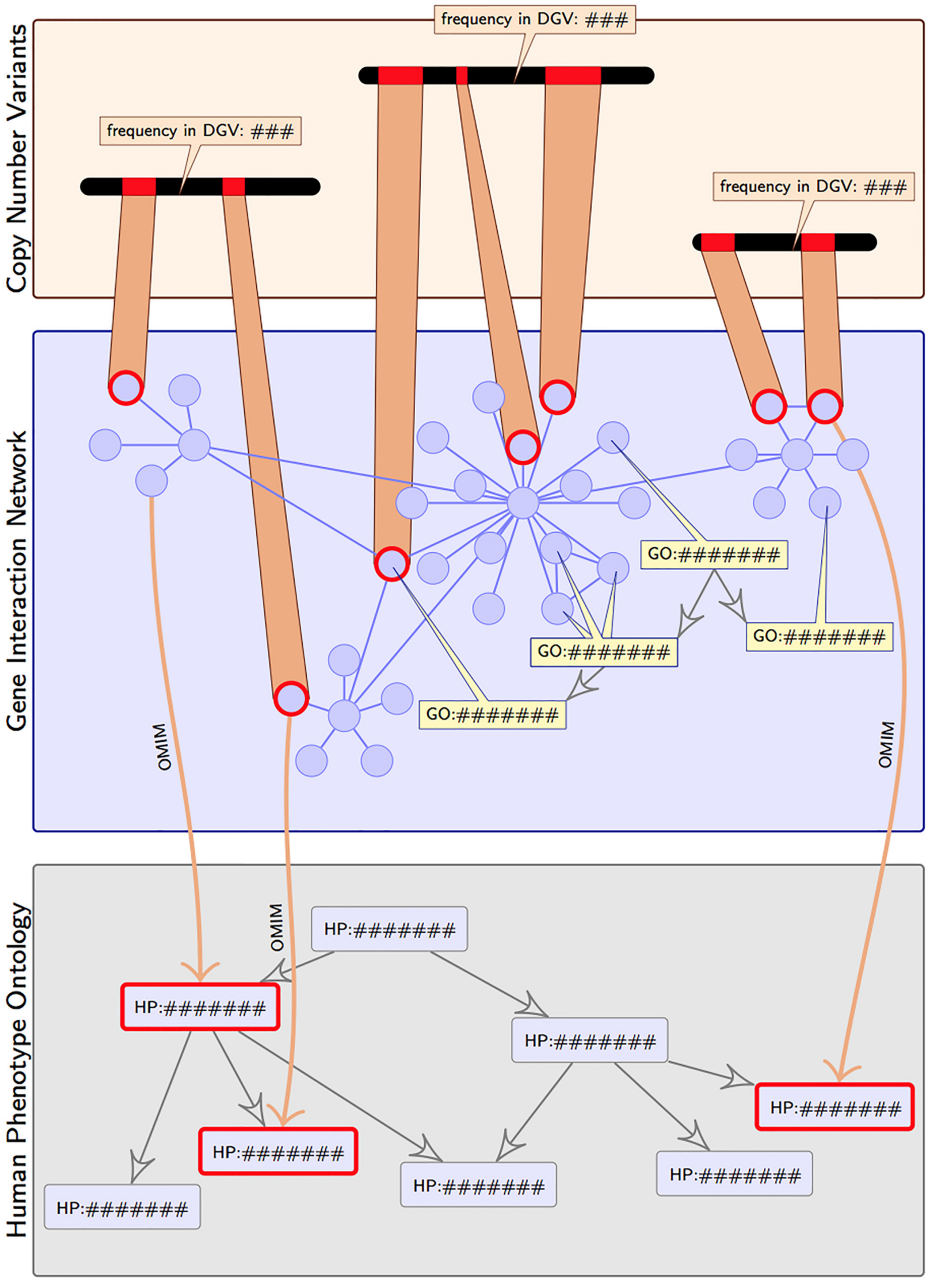
Databases, ontologies and known associations used to identify CNV-phenotype correlations. Our approach integrates 3 types of information: 1) CNVs an their non-exhaustive frequency in healthy individuals, 2) genes and gene interactions, with their respective functions (each gene within a CNV is weighted by its likelihood of contributing to the phenotypes; via semantic similarity within the GO ontology), and 3) phenotypic descriptions and relationships between them as specified by HPO, with their non-exhaustive associations to disease genes (via OMIM). For an individuals variants and known HPO phenotypes, genes affected by these variants are highlighted within the gene interaction network, while the phenotypes are emphasized in the phenotype ontology layer.

To establish whether a CNV and its affected genes could be responsible for the phenotypes observed within a patient, we design a classifier that utilizes multiple types of prior biological knowledge, overviewed in Fig 3. In particular, our method uses phenotype-genotype correlations, gene interaction networks, and gene function to identify genes within each CNV which are most likely to be responsible for the observed patient phenotypes. This gene prediction is then combined with additional information to predict the harmfulness of the CNV.

### 5.1 Classifier Overview

Our method involves using two classifiers: the Gene classifier (see Section 5.2) and the CNV classifier (see Section 5.3). The output of the first results in an input to the second. Table 1 summarizes the features utilized by these classifiers.

**Table 1:** The overall structure of the two layer classifier, with the output of hte Gene Classifier being one of the inputs to the CNV classifier.

Gene Classifier:	Random Forest	
Inputs:		
GO terms	< *Float* >	The “impact” of the gene on each GO term, calculated in 5.2.1.
HPO terms	< *Bool* >	Vector of all phenotypes, with those present in the current individual set to 1
**Output**:	Bool: CausativeGene	Whether this gene is likely causative of the observed phenotypes
CNV Classifier:	Naive Bayes	
Inputs:		
CausativeGene	Bool	The ‘OR’ of CausativeGene status of genes in this CNV from above.
Length	Int	The length of the CNV.
DGV	Float	Score describing whether the CNV is commonly observed in controls (see 5.3).
**Output**:	Harmful/Benign	Harmfulness prediction for the CNV.

Our method is designed to deal with missing data, biases, and other artifacts. An example of missing data is the HPO annotations that relate phenotypes to causative genes. These are likely only a small subset of all existing associations. One bias with gene-gene interactions is that some have been proven experimentally while others are inferred by association, e.g. co-expression. To overcome these issues, we train classifiers to recognize data that is consistent with the provided patient samples.

#### 5.1.1 Train/test split and Experimental setup

For each experiment, we randomly split the CNVs into three sets, which are balanced regarding the representation of each phenotype in the sets and the *harmful:benign* ratio in the training data. The three sets consist of 50% of the CNVs for training the gene classifier, 25% for testing the gene classifier and training the CNV classifier, and 25% for testing the CNV classifier. We test our method on a balanced collection of benign and harmful CNVs. To do so, we iterate through the phenotypes present in the dataset and we select randomly two cases and two controls (CNVs) that are equally distributed into the training and test sets. When it is no longer possible to select two cases and two controls for the two pools, we proceed to the next available phenotype. In certain experiments, we also utilized an “unbalanced” regime, where the above split was based on patients rather than CNVs, and all of the CNVs for a specific patient were assigned to the appropriate benign and harmful categories. This “unbalanced” regime correspond more closely to the clinical setting, where all of the CNVs for a specific patient, the majority of which are benign, are presented for analysis.

All experiments presented in this paper are the aggregation of 20 different runs on random training / testing splits of our initial dataset.

### 5.2 Gene Classification method

The data points used for training and testing the gene classification model are genes affected by the CNVs present in the dataset. A feature vector describes every one of these data points where each feature is a gene function from GO Slim. Since our original data does not comprise any information regarding the actual causative gene for each harmful CNVs, the *harmful / benign* labeling of CNVs from the training set was propagated directly to the underlying genes. In previous subsections we present and evaluate various options for assigning training weights to features (GO terms) and data points (genes) based on known gene-phenotype associations and gene interaction networks.

We used the implementation of Random Forests [29] provided by the Weka software package for Machine Learning [30]. Weka is a tool for data analysis that includes implementations of data pre-processing, classification, regression, clustering, association rules, and visualization by different algorithms. Random forests are a machine learning ensemble method of decision trees. We used random forests because their error rates comparable to other ensemble methods such as AdaBoost [31], while they are more robust to noise [32] which is likely to be introduced by our naive transfer of harmful/benign labels from CNVs to genes.

#### 5.2.1 Feature weighing by incorporating gene interactions

Because we wish that our feature set offer a good overview of the functions affected by any given variant, we expand the GO feature subset to GO terms that annotate genes known to interact with those represented by our data points. We obtain the most relevant neighbors of a gene by *random walk* on a gene network generated by GeneMANIA, and use the GeneMANIA network weights to derive feature weights for the GO terms describing our genes. In this section we propose several feature weighing systems and evaluate their effect on the performance of our model.

We used GeneMANIA’s *Homo sapiens precombined* to create a network of most relevant genes that interact with the ones being investigated. We filtered the network to retain the top 5% of the strongest interactions. We translated the weighted interactions into distances to prepare the graph for Dijkstra’s algorithm. Dijkstra’s algorithm was used to select subnetworks centered on these genes comprising the closest 30 neighbors. This is to restrict the neighbours that we consider to those that are more relevant and to reduce the run time of the random walk. Dijkstra’s algorithm finds the shortest path which corresponds to the closest neighbors due to how we set the distance between nodes to be inversely proportional to the weight of the interaction. To any edge connecting two gene nodes *g*_*i*_ and *g*_*j*_, we assigned a distance *d*_*g*_*i*_, *g*_*j*__ inversely proportional to its weight:

$$\begin{array}{c} d_{g_{i},g_{j}}=\frac{\max_{\left(g_{u},g_{v}\right)}w_{g_{u},g_{v}}}{w_{g_{i},g_{j}}} \end{array}$$ (3)

where max_(*g*_*u*_, *g*_*v*_)_ *w*_*g*_*u*_, *g*_*v*__ is maximum weight found in the entire network. Instead of only using pairwise interactions between genes, we would like to rank the interaction between the query gene and the genes in the subnetwork using information from the whole subnetwork. This is accomplished using a random walk. In each subnetwork of closely related genes, we added a *sink* node adjacent to all genes that had at least one connection removed when generating the subnetwork. A sink node was used to replace the lost connections due to using subnetwork instead of the whole network. The weight of the edge between such a gene and the sink is the sum of the weights of all the connections removed for that gene. We then estimate the strength of interaction between any gene in the subnetwork and the original query gene as the probability distribution of being at any particular node, calculated by a *random walk*, and use these probabilities to rank the closest interactions of a gene. These ranks dictate the order in which we consider the closest neighbors *g*_*n*_ of a query gene *g*_*q*_, with weights inversely proportional to the Dijkstra path distance:

$$\begin{array}{c} w_{g_{q},g_{n}}=\frac{\max_{\left(g_{u},g_{v}\right)}w_{g_{u},g_{v}}}{d_{g_{q},g_{n}}}. \end{array}$$ (4)

Having obtained the most relevant neighboring genes, we extend the original feature set of the target gene by adding all the GO Slim terms that annotate these neighbors as features. Let *g*_*q*_ be a query gene residing in an investigated CNV, and *subnetwork*(*g*_*q*_) denote the weighted graph containing its most relevant neighbors. We have explored several approaches of assigning weights to these features:

**Network-Uniform:** The value 1 is assigned to any GO term feature (*fWeight*_*u*_(*f*) = 1), regardless whether it annotates the affected gene or one of its neighbors.
**Network-Weighted:** The weight of a GO feature *f* is made dependent on the rank *r*_*g*_*q*_, *g*_*n*__ by random walk of the gene neighbor *g*_*n*_ with which it is associated, and the evidence tier level *e*_*f*, *g*_*n*__ associating the GO term *f* to the gene *g*_*q*_:
  $$\begin{array}{c} fWeight_{r}\left(f\right)=\sum_{g_{n}\in subnetwork\left(g_{q}\right)}\frac{1}{10^{r_{g_{q},g_{n}}}}\cdot \frac{1}{10^{e_{f,g_{n}}}} \end{array}$$ (5)
  where, for convenience, we consider *r*_*g*_*q*_, *g*_*q*__ = 0. The evidence tier level *e* associates the value 1 (i.e. more strength) to experimentally proved sources, and 2 to computational evidence sources and other inferred sources. Mappings between genes and GO terms that have no traceable source or no data support are ignored. While evidence codes cannot be used as a measure of the quality of the annotation, we have found that using the above broad categories have improved the algorithm.

#### 5.2.2 Gene weighing via known gene-phenotype associations

In this section we explain the weighing of training data points (genes). This weighting depends on the genes relevance to the associated phenotype, based on previously known gene-phenotype associations.

For each gene *g*_*q*_ affected by a CNV of a patient *q* presenting the phenotypes *P*_*q*_, we compute *relevance*(*g*_*q*_, *P*_*q*_) given by Eq (6). This relevance expresses how likely it is for that gene to be the actual cause of the observed phenotypes *P*_*q*_, based on prior knowledge regarding genes implicated in phenotypes strongly similar to *P*_*q*_. The method computing *relevance*(*g*_*q*_, *P*_*q*_) relies on the similarity between the gene and other genes known to cause phenotypes very similar to *P*_*q*_. The relevance score has two components: the functional similarity of genes and the relatedness of phenotypes. Let *D* = {*e*_*t*_ = (*g*_*t*_, *P*_*t*_)} be the mapping between genes and sets of phenotypes they may produce if mutated. We further labeled each gene with its Gene Ontology functions, as explained in section 3.1.2. Let *GO*(*g*) denote the set of GO terms associated with a gene *g*. Note that at this point each entry (*gene*, *phenotypes*) is expressed as two sets of ontology terms: a set of GO terms describing gene functions and a set of HPO terms describing the observed phenotypes. Then we can compute the score of *g*_*q*_ with respect to the phenotypes *P*_*q*_ as:

$$\begin{array}{c} relevance\left(g_{q},P_{q}\right)=\max_{(g,P)\in D}sim(GO\left(g_{q}\right),GO\left(g\right))\cdot sim\left(P_{q},P\right) \end{array}$$ (6)

where the similarity *sim*(*X*, *Y*) between two sets of ontology terms (either HPO or GO) is based on information content and is given by Eq (1) in Section 2. If this relevance score is zero, we identify the gene’s closest neighbors (see Section 5.2.1) and use the maximum relevance score among the neighbors, scaled by the Dijkstra weight given in Eq (4).

The scores obtained according to Eq (6), which combine the similarity of gene functions with phenotype similarity, ensure that both sources of evidence are present when inferring the possible involvement of a gene affected by a CNV in the observed phenotype. We assign to each of our training data points weights that are proportional to their relevance score, and we expect this procedure to help the classification algorithm focus on genes that are more informative, according to prior knowledge. The weight of a gene *g*_*q*_ affected by a CNV *C* of a patient presenting the phenotypes *P* is calculated as:

$$\begin{array}{c} gWeight_{r}\left(g_{q}\right)=\left\lceil \frac{relevance(g_{q},P)}{\sum_{g_{n}\;\text{affected}\;\text{by}\;C}relevance(g_{n},P)}\cdot 1000\right\rceil . \end{array}$$ (7)

and is interpreted as the number of copies representing each gene in the training set. Note that in this setup each CNV is equally represented in the training set by a total of 1,000 copies of genes. 1,000 genes per CNV was found to give enough granularity to the training data to make the weighting relevant vs the actual number of genes within the CNVs. As an exception, CNVs containing only genes with zero relevance get a single copy per gene, since we consider such genes mostly uninformative and prone to introducing noise in the training set.

In the Results section above, we compare the performance of this **Phenotype-Weighted** model expressed by Eq (7) with the **Phenotype-Uniform** model, where the gene relevance with respect to the given phenotype is not taken into account, and each gene of the CNV has the same number of copies:

$$\begin{array}{c} gWeight_{u}\left(g_{q}\right)=\left\lceil \frac{1}{\#(g_{n}\;\text{affected}\;\text{by}\;C)}\cdot 1000\right\rceil . \end{array}$$ (8)

### 5.3 CNV Classification method

A preliminary analysis of the available data (Fig 1a) revealed that the harmfulness of a CNV is highly correlated with its length, the number of mutated genes and the number of affected gene functions. The unbalanced numbers of affected gene functions between harmful and benign CNVs are likely to lead most classification methods to false correlations between the presence of a certain GO term in the description of the CNV and its harmfulness. Furthermore, treating a CNV as a whole does not make it easy to eventually infer the actual causative gene.

We hence run the Gene Classifier, described above, for each gene within the CNV. The Gene Classifier will decide whether the CNV contains at least one gene that is potentially causative of the observed phenotype. We assign the *causative_gene* feature for any given CNV a true/false value according to this result.

We also use the CNV’s *length* and the CNV’s frequency in the Database of Genomic Varaiants [33] (a large collection of benign variants) as additional features for the CNV classification method. The value of the latter is computed by merging all CNVs within each DGV study to define regions of the genome that we believe are benign. Then, for any given query CNV, we find all of the merged DGV variants that overlap with at least 50% of its length. The sum of the percentage overlap for each of these DGV CNVs is the value assigned to the *DGV_frequecy* feature.

The implementation of Naive Bayes provided by Weka is then trained and tested on CNVs described by these three features. Naive bayes is a machine learning classifier based on Bayes rule, which assumes independence of the features. We chose to use Naive Bayes at this level since the resulting model is easy to interpret and there are only three features.

## Supporting Information

S1 Table Agilent CGH 180k Microarray CNVs. The columns are chromosome, start, end, type [DUP/DEL], phenotype, classification [HARMFUL/BENIGN].
(TXT)
